# Evaluation of Redox Profiles of the Serum and Aqueous Humor in Patients with Primary Open-Angle Glaucoma and Exfoliation Glaucoma

**DOI:** 10.3390/antiox9121305

**Published:** 2020-12-19

**Authors:** Yuji Takayanagi, Yasuyuki Takai, Sachiko Kaidzu, Masaki Tanito

**Affiliations:** Department of Ophthalmology, Shimane University Faculty of Medicine, Izumo 693-8501, Japan; y.takayanagi1008@med.shimane-u.ac.jp (Y.T.); takai611@med.shimane-u.ac.jp (Y.T.); kecha@med.shimane-u.ac.jp (S.K.)

**Keywords:** redox status, oxidative stress, antioxidants, intraocular pressure, primary open-angle glaucoma, exfoliation glaucoma, aqueous humor, superoxide dismutase, diacron reactive oxygen metabolites, biologic antioxidant potential, sulfhydryl test

## Abstract

Oxidative stress is thought to play a significant role in the development of glaucoma. However, the association between systemic and local oxidative stresses in different types of glaucoma has not been assessed fully. The current study compared the redox status in the aqueous humor (AH) and blood samples among eyes with primary open-angle glaucoma (POAG), exfoliation glaucoma (EXG), and non-glaucomatous controls to evaluate the relationship among systemic redox status, intraocular oxidative stress, and clinical backgrounds. AH and blood samples were obtained from 45 eyes of 45 Japanese subjects (15 POAG, 15 EXG, and 15 control eyes). The serum levels of lipid peroxides, ferric-reducing activity, and thiol antioxidant activity were measured by diacron reactive oxygen metabolites (dROM), biologic antioxidant potential (BAP), and sulfhydryl (SH) tests, respectively, using a free radical analyzer. The activities of cytosolic and mitochondrial forms of the superoxide dismutase (SOD) isoforms, i.e., SOD1 and SOD2, respectively, in AH and serum were measured using a multiplex bead immunoassay. In AH, SOD1 in subjects with EXG and SOD2 in those with POAG and EXG were significantly higher than in control eyes. In serum, compared to control subjects, BAP in subjects with POAG and EXG was significantly lower; SOD1 in those with EXG and SOD2 in those with POAG and EXG were significantly higher. dROM and SH did not differ significantly among the groups. The BAP values were correlated negatively with the SOD1 concentrations in AH and serum, SOD2 in the AH, intraocular pressure, and number of antiglaucoma medications. In conclusion, lower systemic antioxidant capacity accompanies up-regulation of higher local antioxidant enzymes, suggesting increased oxidative stress in eyes with OAG, especially in EXG. Determination of the systemic BAP values may help predict the redox status in AH.

## 1. Introduction

Glaucoma, which is a leading cause of irreversible blindness worldwide [1,2], is a multifactorial disease characterized by retinal ganglion cell death [3]. Raised intraocular pressure (IOP) is a major risk factor responsible for the glaucomatous changes in the optic disc [4,5,6,7]; however, the exact mechanism of glaucoma is largely unknown, and raised IOP does not fully explain the development of glaucoma, such as normal tension glaucoma [8,9]. Therefore, the involvement of various genetic [10] and environmental factors, including immune response [11], inflammation [12], ocular blood flow [13,14,15], systemic hypotension [16], and oxidative stress [17,18], have been proposed for the pathogenesis of glaucomatous optic neuropathy.

Oxidative stress is defined as an imbalance between oxidant generation and antioxidant response [19]. The excess production of reactive oxygen species, such as superoxide, can damage a wide variety of biomolecules and induce cellular dysfunction [20], including trabecular meshwork cells. The trabecular dysfunction can obstruct the outflow of the aqueous humor (AH) [17,18], leading to IOP elevation and consequently contributing to retinal ganglion cell death [21]. In contrast, antioxidants can scavenge reactive oxygen and nitrogen species by interacting with various redox signaling pathways [22], which prevents retinal ganglion cell death [23]. Previous studies have demonstrated that low levels of biologic antioxidant potential (BAP), an indicator of the serum antioxidant capacity [24], were related to the development of glaucoma [25]. We previously found that lower systemic BAP levels were associated with higher IOP values in patients with glaucoma and control subjects and with worse visual field defects in open-angle glaucoma (OAG) [26,27]. Oral supplementation of antioxidants might decrease IOP in patients with glaucoma [28]. Overall, this evidence suggested that oxidative stress is likely to play a significant role in the pathogenesis of glaucoma; however, the clinical impact of oxidative stress on the pathogenesis of glaucoma and the relationship between systemic redox status and intraocular ocular oxidative stress in various types of glaucoma are still to be investigated.

The current study compared the redox status in the AH and serum among patients with primary OAG (POAG), exfoliation glaucoma (EXG), and non-glaucomatous controls. We also assessed possible associations between systemic and local redox status and clinical parameters.

## 2. Materials and Methods

### 2.1. Subjects

The current study procedures were carried out in accordance with the Declaration of Helsinki. The institutional review board of Shimane University Hospital approved the research (No. 20091119-1 and No. 20131216-1). All participants provided written informed consent for inclusion in the study. This study included 15 eyes of 15 patients (6 men, 9 women; mean age, 67.0 ± 12.3 years) with POAG (including 1 pseudophakic eye), 15 eyes of 15 patients (10 men, 5 women; mean age, 75.7 ± 5.9 years) with EXG (including 2 pseudophakic eyes), and 15 eyes of 15 patients (6 men, 9 women; mean age, 74.9 ± 9.4 years) with only senile cataracts as controls, who had undergone cataract and/or glaucoma surgery. All surgeries took place at Shimane University Hospital from May 2017 to February 2018. All participants underwent ophthalmologic examinations including measurements of the best-corrected visual acuity (BCVA); IOP measured by Goldmann applanation tonometry; and slit-lamp, gonioscopic, and pupil-dilated funduscopic examinations. Briefly, we diagnosed POAG based on the findings including bilateral open iridocorneal angles and a characteristic appearance of glaucomatous optic neuropathy such as enlargement of the optic disc cup or focal thinning of the neuroretinal rim, corresponding visual field defects detected by the Humphrey Visual Field Analyzer Swedish Interactive Thresholding Algorithm central 30-2 program (Carl Zeiss Meditec, Dublin, CA, USA) in at least one eye, and no evidence of secondary glaucoma bilaterally. We diagnosed EXG in the presence of exfoliation material deposition on the anterior lens capsule and/or at the pupillary border observed by slit-lamp examination, IOP > 21 mmHg, and/or glaucomatous optic neuropathy with consistent visual field changes. The controls had senile cataract, no glaucomatous optic neuropathy, no history of IOP exceeding 21 mmHg, and no use of antiglaucoma medications. Except for a cataract and/or glaucoma, no subjects had ocular problems such as clinically detectable ocular inflammation, infection, neuropathies, retinopathies, or maculopathies.

### 2.2. Collecting Blood and AH Samples

Venous blood samples were collected from the antecubital vein using evacuated tubes [29]. The temperature of samples was maintained at 4 °C during the transportation from the clinic to the laboratory, and during centrifugation of samples. Serum samples were obtained by centrifugation of the collected venous blood, and were stored at 4 °C until the measurement of redox markers. At the beginning of glaucoma or cataract surgery, AH samples (100–200 μL) were obtained by aspiration through a limbal paracentesis using a 0.5-mL syringe with a 30-gauge needle (BD Japan, Tokyo, Japan). During the sampling, care was taken to prevent contamination of blood and intraocular tissues [30]. AH samples were frozen with liquid nitrogen immediately after the sampling, and then stored at −80 °C until the analyses. All patients were prescribed topical 0.5% levofloxacin four times daily for 3 days preoperatively. Patients who experienced cataract surgery or combined cataract and glaucoma surgery were instilled 1% tropicamide, 5% phenylephrine and 0.4% oxybuprocain every 30 min for 2 h prior to the operation. Patients who underwent glaucoma surgery alone were instilled 2% pilocarpine and 0.4% oxybuprocaine every 30 min for 2 h prior to the operation.

### 2.3. Oxidative Stress Measurements

To analyze the serum concentrations of reactive oxygen metabolites, antioxidant capacity, and thiol antioxidant capacity, diacron reactive oxygen metabolite (dROM), we performed BAP and sulfhydryl (SH) tests, respectively. For analyses, we used the free radical analyzer system (FREE Carpe Diem, Wismerll Company Ltd., Tokyo, Japan), which include a spectrophotometric device reader and a thermostatically regulated mini-centrifuge. We performed measurements using the measurement kits that were optimized to the FREE Carpe Diem System, according to the manufacturer’s instructions [29]. All analyses were conducted within 48 h of venous blood sampling to avoid falsely high or low results based on the manufacturer’s recommendation.

The BAP test, measured as its reducing potential against ferric ions, provides an estimate of the global antioxidant capacity of the serum. We measured the intensity of decoloration spectrophotometrically detectable at 505 nm, which occurred when samples are added to a colored solution obtained by mixing a ferric chloride solution and a thiocyanate derivative solution. The measured intensity of decoloration is proportional to the ability of serum to reduce the ferric ions [24,31]. The unit of μmol/L of the reduced ferric ions is adopted for expression of results.

The dROM test evaluates an amount of serum hydroperoxides produced by free radicals. The hydroperoxides in the sample mainly react with transition ions liberated from proteins in an acidic medium and are converted to alkoxy and peroxy radicals. These radicals oxidize an additive aromatic amine (N, N-diethyl-para-phenylendiamine) and form a relatively stable colored cation radical that is spectrophotometrically detectable at 505 nm [32,33]. The arbitrary units (U. Carr), one unit of which corresponds to 0.8 mg/L of hydrogen peroxide [32,33], are adopted for expression of results.

The SH test estimates the total thiol groups in the biologic samples with a modified Ellman method [34,35]. The SH groups in the sample react with 5,5-dithiobis-2-nitrobenzoic acid, which develops a stained complex that is spectrophotometrically detectable at 405 nm. The concentrations of total thiol groups were estimated by the Beer–Lambert law [31,33]. The unit of μmol/L of the SH groups is adopted for expression of results.

The activities of superoxide dismutase (SOD) 1 and SOD2 in the AH and serum were measured using the multiplex bead immunoassay system, MILLIPLEX^TM^ MAP Kit (Merck Millipore, Darmstadt, Germany), according to the manufacturer’s instructions, as we previously reported [30]. SOD1 is a copper- and zinc-dependent form of SOD that is expressed predominantly in the cytosol, and SOD2, a manganese-dependent form of SOD that is expressed predominantly in the mitochondria; SOD1 and SOD2 had been found in serum [36] and SOD1 in AH [37]. Based on the information provided by the manufacturer, the multiplex assay kit can quantitatively measure concentrations of analytes as fluorescent reporter signals from as little as 25 µL of bodily fluids. Minimum detectable concentration is 0.03 and 0.04 ng/mL for SOD1 and SOD2, respectively. Each sample was run as a single measurement for a limited quantity of collected AH. The results are expressed as ng/mL of the SOD groups.

### 2.4. Statistical Analysis

For comparisons among the control, POAG, and EXG groups, the differences in continuous data, i.e., age; BCVA; highest IOP; number of topical antiglaucoma medications; and the levels of SOD1, SOD2, BAP, dROM, and SH, were calculated using the Kruskal–Wallis test and the difference in the categorical datum, i.e., sex, was calculated using the G test. For comparisons between pairs of the control, POAG, or EXG groups, we performed the post-hoc Mann–Whitney U-test for continuous data. *P* values of 0.0167 and 0.0033 for the post-hoc Mann–Whitney U-test were considered significant at 5% and 1%, respectively, based on the Bonferroni correction. Spearman’s correlation test was performed on redox parameters, i.e., SOD1 and SOD2 in AH, and SOD1, SOD2, BAP, dROM, and SH in serum, for which *p* values of 0.05 were considered significant. The correlations between redox parameters, as mentioned previously, and clinical backgrounds, i.e., age, IOP, and number of topical antiglaucoma medications, were assessed using Spearman’s correlation test, for which *p* values of 0.05 were considered statistically significant. We performed all statistical analyses using JMP Pro statistical software version 14.2 (SAS Institute, Inc., Cary, NC, USA). All reported *p* values are two-sided. The data are expressed as the means ± standard deviation (SD) for continuous variables and in numbers and percentage for categorical variables. For the statistical analyses, the decimal BCVA was converted to the logarithm of the minimum angle of resolution (logMAR).

## 3. Results

The demographic subject data, including age, sex, BCVA, IOP, and the number of antiglaucoma medications, among the control, POAG, and EXG groups are shown in Table 1. Age, sex, and BCVA did not differ significantly among the three groups. The IOP was significantly higher in the EXG group (26.4 ± 14.8) than in the control (14.3 ± 3.4; *p* = 0.0012) and POAG (17.1 ± 14.8; *p* = 0.0142) groups; The IOP did not differ between the control and POAG groups (*p* = 0.1042). The number of antiglaucoma medications did not differ between the POAG (2.0 ± 1.5) and EXG (2.9 ± 1.9) groups (*p* = 0.1884).

Table 2 shows the comparisons of various redox parameters among the control, POAG, and EXG groups. In the AH, compared to controls, the mean concentrations of SOD1 and SOD2 were significantly higher in the POAG (*p* = 0.0251 and 0.0062, respectively) and EXG (*p* < 0.0001 both comparisons) groups. In serum, the SOD1 levels were significantly higher in patients with EXG compared to controls (*p* = 0.0137), and the SOD2 levels were significantly higher in patients with POAG (*p* = 0.0062) and EXG (*p* < 0.0001) compared to controls. The mean serum BAP values were significantly lower in patients with POAG and EXG compared with controls (*p* < 0.0001 for both comparisons). The serum dROM and SH concentrations did not differ among the three groups.

The correlations among the redox parameters in the AH and serum are shown in Table 3. Positive correlations were found between SOD1 in the serum and AH (ρ = 0.4167 and *p* = 0.0049). Furthermore, the serum BAP was correlated negatively with both SOD1 (ρ = −0.4485 and *p* = 0.0020) and SOD2 (ρ = −0.5078 and *p* = 0.0004) in the AH, and SOD1 in serum (ρ = −0.3524 and *p* = 0.0190). The dROM and SH levels in the serum were not correlated strongly with the other parameters.

Table 4 shows the possible correlations between seven redox parameters and three clinical parameters including age, IOP, and antiglaucoma medications. SOD1 (ρ = 0.4107 and *p* = 0.0051) and SOD2 (ρ = 0.6596 and *p* < 0.0001) in the AH and SOD1 in serum (ρ = 0.3075 and *p* = 0.0423) were correlated positively with the number of antiglaucoma medications. The serum BAP was significantly negatively correlated with the IOP (ρ = −0.4542 and *p* = 0.0017) and the number of antiglaucoma medications (ρ = −0.6590 and *p* < 0.0001). A relatively weak but significant correlation was found between the serum dROM and IOP (ρ = −0.3096 and *p* = 0.0385) and the number of antiglaucoma medications (ρ = −0.3714 and *p* = 0.0120). In addition, a significant correlation was seen between the serum SH and age (ρ = −0.4898 and *p* = 0.0006).

## 4. Discussion

We compared the redox status both in the AH and serum among patients with POAG and EXG and those without glaucoma and evaluated the relationship among the systemic redox status, intraocular oxidative stress, and clinical backgrounds. Overall, the results suggested three major findings. First, statistically higher SOD species were seen in the AH of patients with POAG and EXG compared with controls. Second, the serum BAP and SOD1 levels were significantly associated with SOD1 in the AH. Third, the serum BAP was correlated negatively with the IOP and the number of antiglaucoma medications. To the best of our knowledge, this is the first study that examined various oxidative stress markers/antioxidative enzymes in serum and aqueous humor by including control, POAG, and EXG subjects, and conducted correlation analyses of various redox parameters and clinical parameters comprehensively.

First, our results showed that SOD species in the AH from patients with POAG and EXG were significantly higher than those from controls. Previous reports have found significant increases in SOD activity in the AH in patients with POAG and EXG compared to non-glaucomatous controls [38,39]. The current results agreed with those of the previous studies, and the evidence we provided suggested that ocular oxidative stress may play an important role in the pathogenesis of EXG and POAG.

It was especially noteworthy that the elevations of SOD1 and SOD2 were markedly higher in EXG than POAG. Previous studies have reported that the serum malondialdehyde levels, the end products of lipid peroxides, were significantly higher in patients with EXG than with POAG [40,41]. Our previous studies have indicated that the enzymatic and singlet oxygen-mediated fatty acid oxidation may be the major oxidation pathways in patients with POAG [42], while free radical-mediated oxidation may be a major oxidation pathway in EXG [43]. Oxidative stress also can enhance production of several cytokines, including interleukins, interferon-gamma, and tumor necrosis factor alpha [44], which contributes to the axonal injury in glaucoma [45], and the excess production of exfoliative material [46,47,48,49]. The SODs are antioxidant enzymes that act as protective agents against oxidative stress; thus, the increase in SOD species in the AH can reflect elevated oxidative stress in glaucomatous eyes, especially in EXG compared with controls. The exact molecular mechanisms to explain these phenomena warrant further research.

The second finding is that the decreased serum BAP level, a reliable marker of systemic antioxidative capacity, and increased serum SOD1, a major subtype of SOD species [50], were correlated significantly with increased SOD1 in the AH. A previous report found that a lower level of systemic BAP was associated with glaucomatous damage in the retinal ganglion cells [25]. We previously found lower BAP values in POAG and EXG compared to controls [29]. Another previous study showed significantly lower total antioxidant status and higher 8-hydroxy-2’-deoxyguanosine, which is a dependable biomarker of oxidative DNA damage, in both the serum and AH obtained from glaucomatous eyes compared with non-glaucomatous eyes [51]. The current evidence suggested that the oxidative stress in the AH might be an ocular manifestation of systemic redox status (i.e., increased oxidative stress due to a decreased antioxidant defense) in patients with glaucoma.

The third current finding to support this hypothesis is that the serum BAP concentrations were strongly negatively correlated with the IOP and the number of antiglaucoma medications. Moreover, the serum dROM levels were correlated weakly, and the SH levels were not correlated with those factors. Our previous research showed that a serum lower BAP level was correlated with higher IOP and severe visual field damage in patients with open-angle glaucoma [26,27]. Those findings agreed partially with the current study and may explain the relevance between the systemic redox status and the glaucomatous changes. The BAP levels indicated the ability to reduce ferric ions (Fe^3+^) to ferrous ions (Fe^2+^), and reduced BAP values mean increased oxidative stress in human cells [24,31]. The main component detected by the dROM test is hydroperoxide, which also induces oxidative stress in tissue, leading to cellular damage and death [52]. Therefore, the homeostasis between BAP and dROM values may be implicated in the oxidative stress-induced cellular damage in the trabecular meshwork cells, which may explain the correlations between these oxidative stress levels and IOP elevation. Overall, the current study indicated that intra-ocular oxidative stress triggered by systemic oxidative stress such as lower BAP might be a causal factor for glaucoma.

It is interesting to note that the SH test measured the total thiol levels in the serum [34,35], which were strongly correlated with age and not with the IOP in the current study. Our previous research also showed a strong negative correlation between the SH levels and age [29], which was consistent with the current results. Other previous research indicated that decreased glutathione and cysteinylglycine levels with the concomitant increase of oxidized thiols were correlated with age [53]. Other reports have indicated that serum free thiols may play an important role in protecting tissue against oxidative stress and the thiol/disulfide balance is a key protective factor, rather than the total thiol concentration [54,55,56]. Nevertheless, we did not evaluate the serum free thiols in this study, and this requires further clarification.

It is also important to emphasize that the number of antiglaucoma medications was correlated with the SOD species in the AH and the BAP in the serum. Previous reports have shown that topical antiglaucoma medications, especially benzalkonium chloride-preserved eye drops, increased oxidative stress in tear film [57,58]; therefore, our results might reflect oxidative stress induced by topical antiglaucoma treatment. Since the type of antiglaucoma medications was not considered in this study, our findings should be interpreted with caution. However, since the topically applied medications are unlikely to reduce the systemic BAP, elevation of SOD in AH is likely to reflect the severity of glaucoma rather than the direct effect of medications.

Finally, the current study had limitations that may affect the generalization of our findings. First, our study had the same limitations as any retrospective study in that it was neither controlled nor randomized. Second, the small sample size had insufficient power to detect a significant association among all parameters. Third, the controls were selected from those patients who had undergone cataract surgery, which creates a potential selection bias. Despite these limitations, our study has many strengths, including use of both blood samples and AH, a clinically generalizable sample of patients, and comprehensive assessments of patients’ systemic and ocular redox parameters.

## 5. Conclusions

In conclusion, the current study suggested that systemic redox status and ocular oxidative stress were associated significantly with EXG and POAG. Further, the systemic redox parameters were correlated strongly with oxidative stress in the AH, and the systemic antioxidant capacity was significantly negatively correlated with the IOP and glaucoma medication score. This study highlights the clinical utility of systemic antioxidant treatment for patients with glaucoma, in particular, elevated IOP-induced glaucoma such as POAG and EXG. The findings warrant further research to explore the underlying mechanisms of the oxidative stress response in glaucomatous patients and to investigate the effectiveness of antioxidative treatment for glaucoma.

## Figures and Tables

**Table 1:** Demographic subject data.

	Control	POAG	EXG	*p* Value ^a^
N	15	15	15	
Age (years)				
Mean ± SD	74.9 ± 9.4	67.0 ± 12.3	75.7 ± 5.9	0.0781
Range	60–91	48–86	66–86	
Sex				
Men, *n* (%)	6 (40.0)	6 (40.0)	10 (66.7)	0.2410
Women, *n* (%)	9 (60.0)	9 (60.0)	5 (33.3)	
BCVA (logMAR)				
Mean ± SD	0.28 ± 0.28	0.11 ± 0.23	0.12 ± 0.24	0.1357
Range	−0.08–0.82	−0.08–0.70	−0.18–0.7	
Highest IOP (mmHg)				
Mean ± SD	14.3 ± 3.4	17.1 ± 14.8	26.4 ± 14.8	0.0016 **
Range	9.0–20.0	9.0–30.0	12.0–69.0	
*p* value, vs. control ^b^	-	0.1042	0.0012 ^##^	
*p* value, vs. POAG ^b^	-	-	0.0142 ^#^	
Antiglaucoma medications, n (%)				
Mean ± SD	0.0 ± 0.0	2.0± 1.5	2.9 ± 1.9	<0.0001 **
Range	0.0–0.0	0.0–4.0	0.0–7.0	
*p* value, vs. control ^b^	-	<0.0001 ^##^	<0.0001 ^##^	
*p* value, vs. POAG ^b^	-	-	0.1884	

^a^ Comparisons among the control, POAG, and EXG groups using the Kruskal–Wallis test for continuous data and the G test for categorical data. ** indicates the significance levels at 1% (*p* < 0.01). ^b^ Comparisons between either pair of control, POAG, or EXG groups using the post-hoc Mann–Whitney U-test for continuous data. ^#^ and ^##^ indicate the significance levels at 5% (*p* < 0.0167) and 1% (*p* < 0.0033), respectively, by the Bonferroni correction for multiple comparisons. N, number of participants; SD, standard deviation; POAG, primary open-angle glaucoma; EXG, exfoliation glaucoma; BCVA, best-corrected visual acuity; logMAR, logarithm of the minimum angle of resolution; IOP, intraocular pressure.

**Table 2:** Redox status among control, POAG, and EXG groups.

	Control	POAG	EXG	*p* Value ^a^
N	15	15	15	
ah-SOD1 (ng/mL)				
Mean ± SD	25.9 ± 8.1	44.6 ± 25.6	179.4 ± 192.4	0.0001 **
Range	16.9–42.1	16.0–89.6	22.5–571.9	
*p* value, vs. control ^b^	-	0.0251	<0.0001 ^##^	
*p* value, vs. POAG ^b^	-	-	0.0161 ^#^	
ah-SOD2 (ng/mL)				
Mean ± SD	0.42 ± 0.14	0.87 ± 0.63	1.58 ± 2.17	<0.0001 **
Range	0.20–0.71	0.22–2.77	0.67–9.36	
*p* value, vs. control ^b^	-	0.0062 ^#^	<0.0001 ^##^	
*p* value, vs. POAG ^b^	-	-	0.0380	
s-SOD1 (ng/mL)				
Mean ± SD	202.9 ± 70.0	347.9 ± 321.1	381.8 ± 244.1	0.0421 *
Range	87.1–311.8	122.8–1433.1	117.6–956.0	
*p* value, vs. control ^b^	-	0.1102	0.0137 ^#^	
*p* value, vs. POAG ^b^	-	-	0.4450	
s-SOD2 (ng/mL)				
Mean ± SD	0.42 ± 0.14	0.87 ± 0.63	1.58 ± 2.17	<0.0001 **
Range	0.20–0.71	0.22–2.77	0.67–9.36	
*p* value, vs. control ^b^	-	0.0062 ^#^	<0.0001 ^##^	
*p* value, vs. POAG ^b^	-	-	0.0380	
s-BAP (μmol/L)				
Mean ± SD	2996.6 ± 346.0	1881.0 ± 226.1	1895.1 ± 292.8	<0.0001 **
Range	2082.3–3653.0	1525.6–2302.5	1412.7–2303.6	
*p* value, vs. control ^b^	-	<0.0001 ^##^	<0.0001 ^##^	
*p* value, vs. POAG ^b^	-	-	0.8035	
s-dROM (U. Carr)				
Mean ± SD	352.9 ± 64.1	320.5 ± 66.9	331.9 ± 69.1	0.3617
Range	273.0–514.0	217.0–437.0	237.0–505.0	
s-SH (U. Carr)				
Mean ± SD	622.4 ± 100.4	622.9 ± 670.2	613.9 ± 77.5	0.7656
Range	364.0–758.0	452.0–847.0	470.0–733.0	

^a^ Comparisons among the control, POAG, and EXG groups using the Kruskal–Wallis test for continuous data. * and ** indicate the significance levels at 5% (*p* < 0.05) and 1% (*p* < 0.01), respectively. ^b^ Comparisons between either pair of the control, POAG, or EXG groups using the post-hoc Mann–Whitney U-test. ^#^ and ^##^ indicate the significance levels at 5% (*p* < 0.0167) and 1% (*p* < 0.0033), respectively, by Bonferroni correction for multiple comparisons. N, number of participants; SD, standard deviation; POAG, primary open-angle glaucoma; EXG, exfoliation glaucoma; ah, aqueous humor; s, serum; SOD, superoxide dismutase; BAP, biologic antioxidant potential; dROM, diacron reactive oxygen metabolites; SH, sulfhydryl.

**Table 3:** Correlations among systemic and intraocular redox parameters.

ρ/*p* Value	ah-SOD1	ah-SOD2	s-SOD1	s-SOD2	s-BAP	s-dROM	s-SH
ah-SOD1	-	0.6214	0.4167	−0.0869	−0.4485	0.0712	−0.1785
ah-SOD2	<0.0001 *	-	0.2855	0.2042	−0.5078	−0.1468	−0.1666
s-SOD1	0.0049 *	0.0603	-	−0.0078	−0.3524	−0.1938	−0.1933
s-SOD2	0.5703	0.1784	0.9602	-	−0.0917	0.0317	−0.1176
s-BAP	0.0020 *	0.0004 *	0.0190 *	0.5491	-	0.2068	−0.0148
s-dROM	0.6419	0.3358	0.2075	0.8090	0.1729	-	0.0544
s-SH	0.2408	0.2741	0.2088	0.4417	0.9230	0.7225	-

Correlation coefficient (ρ) and *p* values for each pair of groups were calculated using Spearman’s correlation test. * indicates the significance levels at 5% (*p* < 0.05). Ah, aqueous humor; s, serum; SOD; superoxide dismutase, SOD; superoxide dismutase; BAP, biologic antioxidant potential; dROM, diacron reactive oxygen metabolites; SH, sulfhydryl.

**Table 4:** Correlations among redox parameters and clinical parameters.

	Age (Years)		IOP (mm Hg)		Antiglaucoma Medication (n)	
	ρ	*p* Value	ρ	*p* Value	ρ	*p* Value
ah-SOD1	0.1758	0.2480	0.2061	0.1743	0.4107	0.0051 *
ah-SOD2	0.1347	0.3777	0.2783	0.0641	0.6596	<0.0001 *
s-SOD1	0.0067	0.9654	0.1741	0.2584	0.3075	0.0423 *
s-SOD2	0.0310	0.8398	−0.0965	0.5283	0.2407	0.1112
s-BAP	0.1806	0.2351	−0.4542	0.0017 *	−0.6590	<0.0001 *
s-dROM	0.1833	0.2281	−0.3096	0.0385 *	−0.3714	0.0120 *
s-SH	−0.4898	0.0006 *	0.1483	0.3311	−0.0535	0.7270

Correlation coefficient (ρ) and *p* values for each pair of groups were calculated using the Spearman’s correlation test. * indicates the significance levels at 5% (*p* < 0.05). N, number; IOP; intraocular pressure, ah, aqueous humor; s, serum; SOD; superoxide dismutase, SOD; superoxide dismutase; BAP, biologic antioxidant potential; dROM, diacron reactive oxygen metabolites; SH, sulfhydryl.
